# Vitamin C injection improves antioxidant stress capacity through regulating blood metabolism in post-transit yak

**DOI:** 10.1038/s41598-023-36779-w

**Published:** 2023-06-23

**Authors:** Li Zhang, Yi Chen, Ziyao Zhou, Zhiyu Wang, Lin Fu, Lijun Zhang, Changhui Xu, Juan J. Loor, Gaofu Wang, Tao Zhang, Xianwen Dong

**Affiliations:** 1 Chongqing Academy of Animal Sciences, Rongchang, 402460 China; 2 Chongqing Engineering Laboratory of Nano/Micro Biomedical Detection; Chongqing Key Laboratory of Nano/Micro Composite Material and Device, College of Metallurgy and Materials Engineering, Chongqing University of Science and Technology, Chongqing, 401331 China; 3 The Key Laboratory of Animal Disease and Human Health of Sichuan Province, College of Veterinary Medicine, Sichuan Agricultural University, Chengdu, 611130 China; 4 Tibet Leowuqi Animal Husbandry Station, Changdu Tibet, 855600 China; 5 Mammalian NutriPhysioGenomics, Department of Animal Sciences and Division of Nutritional Sciences, University of Illinois, Urbana, 61801 USA

**Keywords:** Biochemistry, Biological techniques

## Abstract

Transportation stress is one of the most serious issues in the management of yak. Previous studies have demonstrated that transport stress is caused by a pro-oxidant state in the animal resulting from an imbalance between pro-oxidant and antioxidant status. In this context, vitamin C has the ability to regulate reactive oxygen species (ROS) synthesis and alleviate oxidative stress. Although this effect of vitamin C is useful in pigs, goats and cattle, the effect of vitamin C on the mitigation of transport stress in yaks is still unclear. The purpose of this study was to better assess the metabolic changes induced by the action of vitamin C in yaks under transportation stress, and whether these changes can influence antioxidant status. After the yaks arrived at the farm, control or baseline blood samples were collected immediately through the jugular vein (VC_CON). Then, 100 mg/kg VC was injected intramuscularly, and blood samples were collected on the 10th day before feeding in the morning (VC). Relative to the control group, the VC injection group had higher levels of VC. Compared with VC_CON, VC injection significantly (*P* < 0.05) decreased the blood concentrations of ALT, AST, T-Bil, D-Bil, IDBIL, UREA, CRP and LDH. However, VC injection led to greater (*P* < 0.05) AST/ALT and CREA-S relative to VC_CON. There was no difference (*P* > 0.05) in GGT, ALP, TBA, TP, ALBII, GLO, A/G, TC, TG, HDL-C, LDL-C, GLU and l-lactate between VC_CON and VC. The injection of VC led to greater (*P* < 0.05) concentration of MDA, but did not alter (*P* > 0.05) the serum concentrations of LPO and ROS. The injection of VC led to greater (*P* < 0.05) serum concentrations of POD, CAT and GSH-PX. In contrast, lower (*P* < 0.05) serum concentrations of SOD, POD and TPX were observed in VC relative to VC_CON. No difference (*P* > 0.05) in GSH, GSH-ST and GR was observed between VC_CON and VC. Compared with the control group, metabolomics using liquid chromatography tandem–mass spectrometry identified 156 differential metabolites with *P* < 0.05 and a variable importance in projection (VIP) score > 1.5 in the VC injection group. The injection of VC resulted in significant changes to the intracellular amino acid metabolism of glutathione, glutamate, cysteine, methionine, glycine, phenylalanine, tyrosine, tryptophan, alanine and aspartate. Overall, our study indicated that VC injections were able to modulate antioxidant levels by affecting metabolism to resist oxidative stress generated during transport.

## Introduction

Yak (*Bos grunnien*) is a unique breed of bovine that can adapt to the Qinghai-Tibet Plateau's (QTP) distinctive and severe natural environment^1^. About 90% of the world's yaks live in China at an altitude of 3500–5000 m all year round. Yak are important to herdsmen in the QTP for providing daily necessities such as meat, milk, wool, skin, draught and fuel^2–4^. However, the severe natural environment often results in feed shortage^5^, often resulting in marked live-weight loss especially during the winter-spring period^6^. Thus, transporting yak to low altitude districts with abundant feed resources for fattening is a suitable strategy to promote sustainability of the yak industry. Oxidative stress might be induced during transit and may damage the health of yak and reduce production performance.

Transportation stress is induced by fasting, capture, vibration, collision, scraping, environmental changes, turbulence and psychological pressure during transport^7^. It has been demonstrated that transportation stress in yak could lead to poor health, immune dysfunction, morbidity, mortality, reduced production performance and product quality, all of which result in huge economic losses^8–10^. Thus, way to reduce transport stress in the yak are urgently needed.

Previous studies confirmed that transportation stress damage is derived from oxidative stress, which is defined as the unbalance between pro-oxidants and antioxidants in the body^11–13^. Vitamin C (VC), as a potent antioxidant, regulates the synthesis of reactive oxygen species (ROS) and alleviates oxidative stress damage^8,9^. This vitamin has been widely used to relieve transportation stress damage in pigs, goats and cattle^14–17^. Furthermore, VC can elicit effects on metabolism at the cellular level^18^. Previous studies reported that VC could alter the synthesis of glutathione (GSH) through regulation of glucose metabolism, amino acid metabolism and energy metabolism^19,20^. GSH is the most potent cellular antioxidant^21^, but to our knowledge, whether VC can elicit antioxidant effects in yak after transportation is unknown.

Metabolomics uses gas chromatography–mass spectrometry (GC–MS), liquid chromatography-tandem–mass spectrometry (LC–MS), and nuclear magnetic resonance (NMR) to elucidate the changes in metabolites and compounds produced by low molecular weight cells and tissues^22^. Recently, metabolomics has been used to study the changes in metabolites in bovine gastric juice, serum, and urine, aiming to identify unknown biomarkers and particular metabolic pathways related to bovine diseases to ensure the health of cattle and reduce economic damage^23^. Our previous research has confirmed that injecting vitamin E into yaks can enhance their ability to resist oxidative stress by inducing changes in metabolites, such as changes in α-Oxo-glutarate, phenylalanine, choline, and malate^24^. To our knowledge, metabolomics has been widely used to study various animal diseases, but the effect of VC on yaks still needs to be determined.

Given the severe natural environment and bad rudimentary facilities of farms in the QTP, from a management standpoint, it is more effective to implement anti-transportation stress treatments in yak after arrival at the destination feedlot. Thus, our hypothesis was that VC could alleviate long-distance transportation stress and accelerate physical recovery once the yaks arrive at the destination. To address this hypothesis, yaks were injected VC immediately after arriving at the destination farm, and then the concentration of VC in blood serum, blood biochemical indices and blood metabolomics were performed to understand in more depth mechanism whereby VC helps alleviate transportation stress in yak.

## Materials and methods

### Animal experiment and sample collection

Five yaks (age, 4 years) were procured, with the initial average weight of 145 kg. They were treated for ecto- and endo-parasites before the start of the experiment and then subsequently at regular intervals. All yaks were transported on asphalt roads at an approximate speed of 60 km/h from Riwoqi County, Tibet Autonomous Region (altitude of approximately 3900 m), to Rongchang District, Chongqing (altitude of 400 m), a trip of approximately 34 h (2100 km). After arriving at the farm, a control blood sample was collected immediately from the jugular vein (VC_CON). Then, 100 mg/kg VC was injected intramuscularly, and the total quantity of VC injected for each yak was 14.5 g, after that blood samples collected as the VC treatment on the 10th day before feeding in the morning (VC). Serum was extracted by centrifugation at 3000 rpm for 10 min at 4 °C and immediately stored at − 80 °C until biochemical and antioxidant index determination and metabolomicse analysis. All yaks were fed the same diet twice daily at 8:30 AM and 5:00 PM. Yaks were given access to drinking water ad-libitum.

### Blood biochemical indices determinations

Blood biochemical assays were performed in an automatic biochemical analyzer (Beckman Coulter AU680). Briefly, all assays were performed using a colorimetric assay (modified kinetic Jaffe method), turbidimetry, latex agglutination, homogeneous EIA and indirect ISE in a Beckman Coulter AU680 analyzer using appropriate commercial test kits.

### Antioxidant indices determinations

The levels or activities of vitamin C (VC), lipid peroxide (LPO), malondialdehyde (MDA), reactive oxygen species (ROS), superoxide dismutase (SOD), peroxidase (POD), catalase (CAT), thioredoxin peroxidase (TPX), glutathione reductase (GR), glutathione S-transferase (GSH-ST), according to the specifications provided by the reagent company, Glutathione peroxidase (GSH-PX) and glutathione (GSH) in serum were measured with a detection kit (Jiancheng Bioengineering Institute, Nanjing, Jiangsu, China).

### Metabolite extraction

A total of 20 μL of sample was transferred to an EP tube and spun for 30 s, sonicated in an ice-water bath for 10 min, and incubated at − 40 °C for 1 h to precipitate the proteins after adding 80 μL of extraction solution (acetonitrile: methanol = 1:1, containing isotopically labeled internal standard mixture). Subsequently, samples were centrifuged at 12,000 rpm for 15 min at 4 °C (RCF = 13,800 (g), R = 8.6 cm). The supernatant that resulted was transferred to a new glass vial for analysis. QC samples were created by combining equal aliquots of supernatant from each sample.

### UHPLC-MS–MS analysis

An UHPLC system (Vanquish, Thermo Fisher Scientific) with an UPLC BEH amide column (2.1 mm 100 mm, 1.7 m) coupled to a Q Exactive HFX mass spectrometer (Orbitrap MS, Thermal) was used for the LC–MS/MS analysis. The mobile phase contained 25 mmol/L ammonium acetate and 25 mmol/L ammonia (pH 9.75). The autosampler was set to 4 °C with a 2 L injection volume. The QE HFX mass spectrometer was chosen because of its ability to obtain MS/MS spectra using the acquisition software's information-dependent acquisition (IDA) mode (Xcalibur, Thermo). In this mode, the acquisition program continuously evaluates the entire MS spectrum scanned. The ESI source conditions listed below were used: Sheath gas flow of 30 Arb, auxiliary gas flow of 25 Arb, capillary temperature of 350 °C, full MS resolution of 60,000.

### Data analysis

First, metabolite features were detected in < 20% of experimental samples or in < 50% of QC samples, they were removed from data analysis. Then, any missing raw data were assigned a value of half of the minimum value. In addition, an internal standard normalization method was employed. Lastlly, features with RSD > 30% were removed from subsequent analysis. The resulting three-dimensional data involving the peak number, sample name, and normalized peak area were fed to the R package metaX for principal component analysis (PCA) and orthogonal projections to latent structures-discriminate analysis (OPLS-DA).

First, metabolite features detected in 20% of experimental samples or 50% of quality control samples were excluded from the data analysis. Then, any missing raw data were assigned a value of half of the minimum value. In addition, an internal standard normalization method was employed. Lastly, features with RSD greater than 30% were excluded from subsequent analyses. The 3D data generated, which included peak number, sample name, and normalized peak area, was fed into the R package metaX for PCA and orthogonal projection to latent structure discrimination analysis (OPLS-DA)^15^. To further demonstrate the model's reliability, the order of the categorical variable Y was randomly changed by a permutation test of 200 times. The t-test and fold-change analysis of data were used to detect and identify differential metabolites between the control and injection groups. In this study, the value criteria were that the p-value of the Student’s t-test was less than 0.05 and the variable importance in projection (VIP) was greater than 1. To visualize metabolites with the same differences by volcano plot, the ggplot2 software package in the R software was used. For metabolic pathway analysis, commercial databases such as KEGG (http://www.kegg.jp) and Metabolic Analysis System (http://www.metaboanalyst.ca/) were used.

SAS (version 9.3; SAS Institute Inc, Cary, NC, USA) was used to analyze data for blood biochemical and blood antioxidant parameters using a mixed model with VC injection as a fixed effect and animals (yaks) as a random effect. The LSMEANS option was used to obtain treatment means, which were then separated using the PDIFF option, with a significance level of *p* < 0.05. Data are presented as means ± standard deviation.

### Ethical approval

This study was conducted in accordance with the administration regulations of experimental animals. The procedure involving animals in this experiment was approved by the ethics committee of the Chongqing Academy of animal sciences (Approval Number: xky‐20180716). This reporting in the manuscript follows the recommendations in the ARRIVE guidelines.

## Results

### Vitamin C concentration in serum

**Figure 1:**
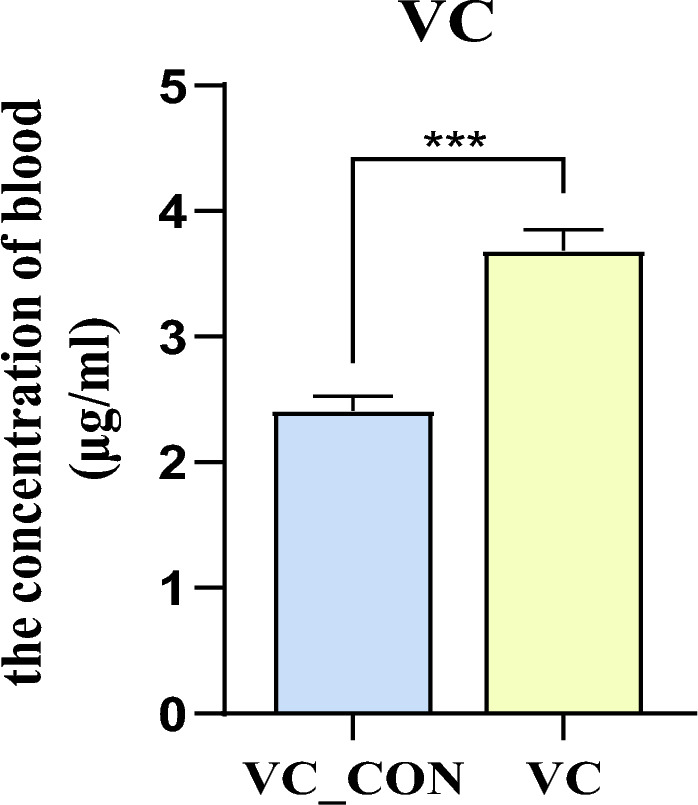
Effects of Vitamin C intramuscular injection on blood concentration of VC in yak.

The effect of VC injection on the blood serum VC level is shown in Fig. 1. Compared with the control (VC_CON), injection significantly (*P* < 0.001) increased the blood serum concentration of VC.

### Blood parameters

The effects of VC on blood biochemical indices are shown in Table 1. A total of 23 biomarkers were detected in this study. Compared with VC_CON, VC injection significantly (*P* < 0.05) decreased the blood concentrations of ALT, AST, T-Bil, D-Bil, IDBIL, UREA, CRP and LDH. However, VC injection led to greater (*P* < 0.05) AST/ALT and CREA-S relative to VC_CON. There was no difference (*P* > 0.05) in GGT, ALP, TBA, TP, ALBII, GLO, A/G, TC, TG, HDL-C, LDL-C, GLU and l-lactate between VC_CON and VC.

**Table 1:** Effect of Vitamin C injection on blood biochemical indexes of Yak.

Items	Treatment		SEM	*P*-Value
	VC_CON	VC		
ALT(U/L)	42.60^a^	24.60^b^	2.71	< 0.01
AST(U/L)	115.00^a^	70.00^b^	8.27	< 0.01
GGT(U/L)	9.80	7.80	0.85	0.19
ALP(U/L)	125.40	100.60	16.54	0.19
AST/ALT	2.67^b^	2.88^a^	0.14	0.01
TBA	12.06	17.88	2.67	0.21
TP(g/L)	68.58	65.22	1.82	0.45
ALBII(g/L)	40.44^a^	36.72^b^	0.88	0.01
GLO(g/L)	28.14	28.70	1.37	0.60
A/G	1.44	1.30	0.06	0.08
T-Bil(μmol/L)	14.02^a^	4.84^b^	0.86	< 0.01
D-Bil(μmol/L)	5.78^a^	1.14^b^	0.44	< 0.01
IDBIL(μmol/L)	8.24^a^	3.70^b^	0.49	< 0.01
UREA(mmol/L)	9.26^a^	5.09^b^	0.38	< 0.01
CREA(μmol/L)	152.08^b^	178.50^a^	6.65	< 0.01
TC(mmol/L)	2.15	2.24	0.12	0.60
TG(mmol/L)	0.22^b^	0.24^a^	0.03	0.07
HDL-C(mmol/L)	1.44	1.47	0.07	0.12
LDL-C(mmol/L)	0.61	0.68	0.06	0.75
CRP(mg/L)	1.53^a^	0.74^b^	0.26	0.07
GLU(mmol/L)	3.44	3.42	0.24	0.38
l-lactate detection (mmol/L)	2.60	3.56	0.42	0.17
LDH(U/L)	867.40^a^	706.67^b^	44.48	0.05

*ALT* glutamate pyruvic transaminase, *AST* Aspartate aminotransferase, *GGT* glutamyl transferase, *ALP* alkaline phosphatase, *TBA* total bile acid, *TP* total protein, *ALBII* albumin, *GLO* globulin, *T-Bil* total bilirubin, *D-Bil* direct bilirubin, *IDBIL* indirect bilirubin, *CHE* cholinesterase, *CREA* creatinine, *TC* total cholesterol, *TG* triglyceride, *HDL-C* high density lipoprotein, *LDL-C* low density lipoprotein, *CRP* C-reactive protein, *GLU* glucose, *LDH* lactate dehydrogenase.

^a,b^Letters means within a row that do not have a common superscript letter differ, *P* < 0.05.

### Oxidative stress state in yak

**Figure 2:**
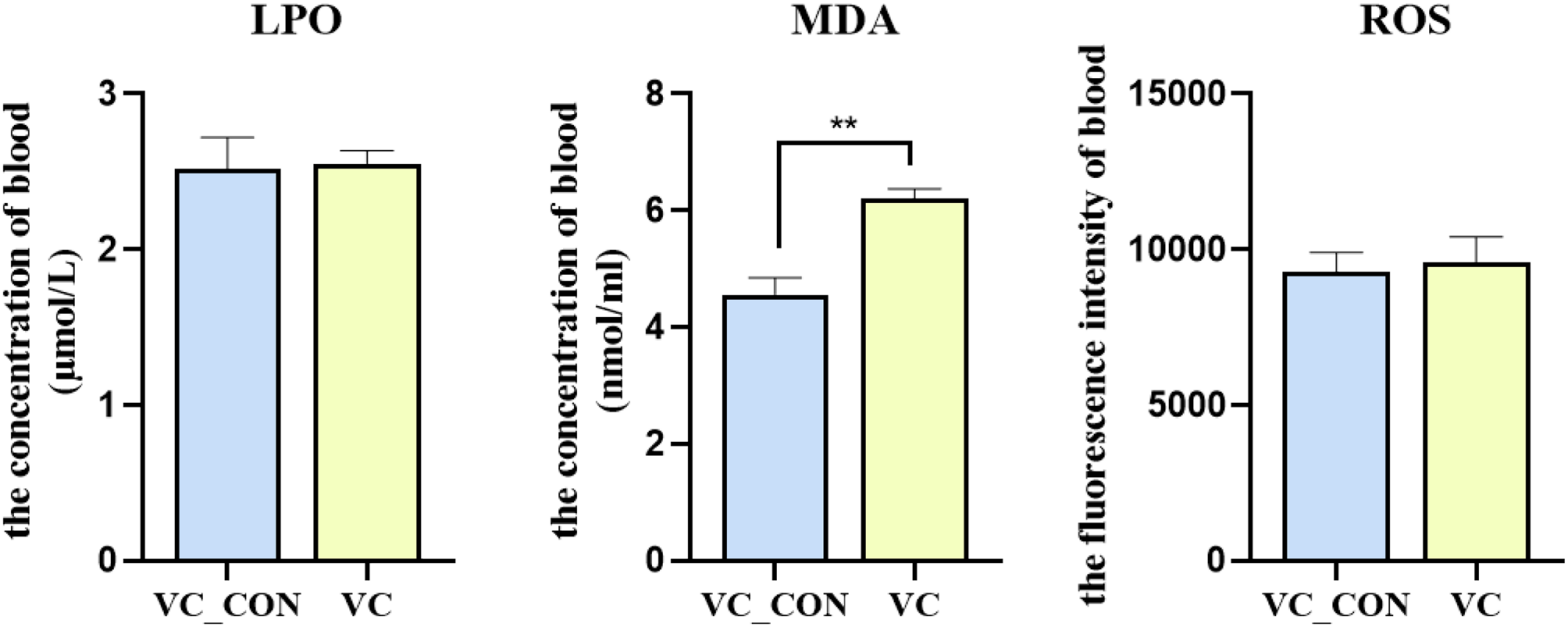
Effects of Vitamin C intramuscular injection on blood serum level of LPO, MDA and ROS in yak.

The effect of VC injection on the serum level of LPO, MDA and ROS level in post-transit yak is shown in Fig. 2. Compared with VC_CON, a greater (*P* < 0.05) concentration of MDA was observed in the VC group. However, VC injection did not alter (*P* > 0.05) the serum concentrations of LPO and ROS.

### Evaluation of anti-oxidant ability

**Figure 3:**
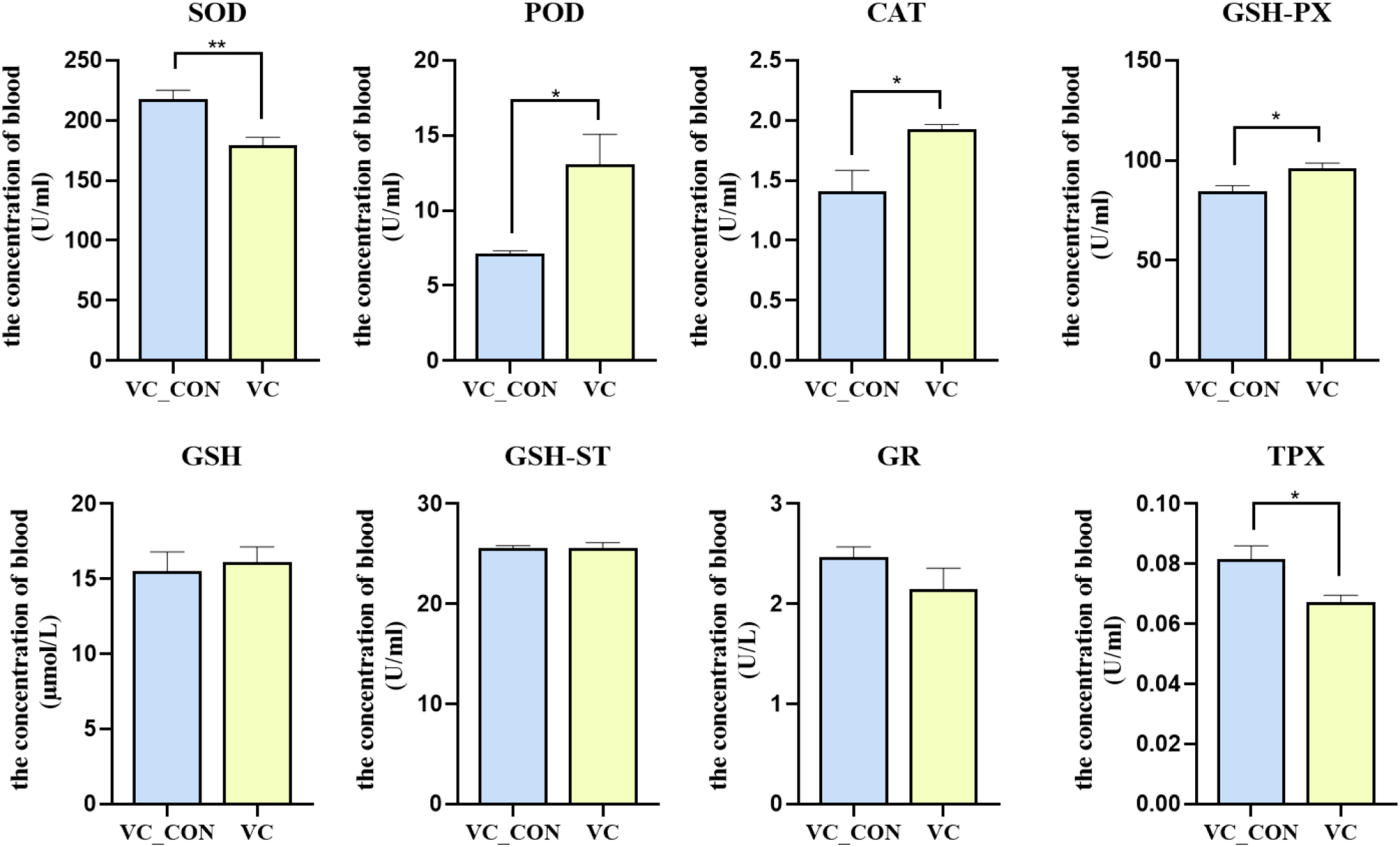
Effects of Vitamin C intramuscular injection on blood serum level of SOD, POD, CAT, GSH-PX, GSH, GSH-ST, GR and TPX in yak.

The effect of VC injection on the blood serum SOD, POD, CAT, GSH-PX, GSH, GSH-ST, GR and TPX level in yak is shown in Fig. 3. Compared with VC_CON, VC led to greater (*P* < 0.05) serum concentrations of POD, CAT and GSH-PX. However, a lower (*P* < 0.05) serum concentration of SOD, POD and TPX was observed in the VC group relative to VC_CON. No difference (*P* > 0.05) in GSH, GSH-ST and GR was observed between VC_CON and VC.

### Metabolomics analysis of serum

**Figure 4:**
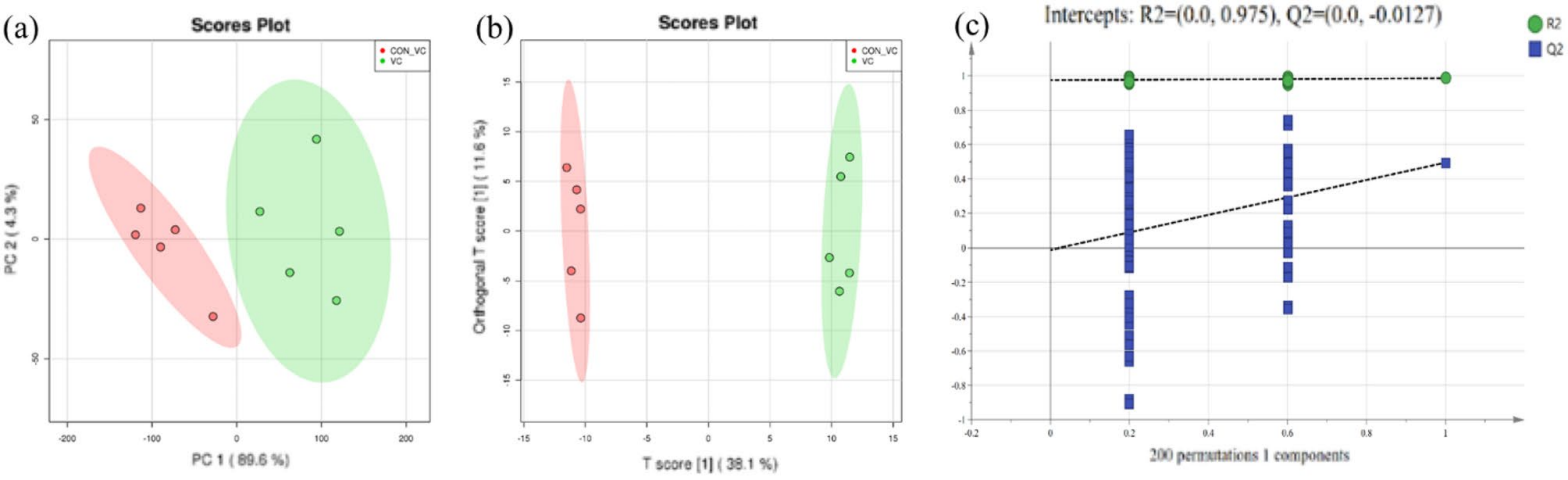
Metabolomics of PCA analysis (**a**), OPLS-DA analysis (**b**) and permutation test of OPLS-DA (**c**). VC_CON = blood sample collected once upon arriving at the farm before injection. VC = blood sample collected after injection on the 10th day after arrival. PCA = principal component analysis, the red represents VC_CON, and the green VC injection. OPLS-DA = orthogonal partial least squares discriminant analysis, the red represents VC_CON, and the green VC injection.

A total of 343 compounds were identified and quantified in serum based on LC–MS/MS analysis (Supplemental Table S1). The multivariate analysis of PCA and OPLS-DA revealed separate clusters between the VC_CON and VC groups (Fig. 4a,b). The parameters of R^2^Y and Q^2^ are both used to evaluate the reliability and predictive ability of the model in OPLS-DA analysis. The R^2^Y was greater than 0.961 suggesting good reliability of the model used in this study. The Q^2^ was greater than 0.898 suggestingt good predictive ability of the model used. A 200 permutation test was performed to avoid overfitting the OPLS-DA model. Both *p*R^2^Y and *p*Q^2^ were less than 1.0, which tindicated good robustness and validity of the model (Fig. 4c).

**Figure 5:**
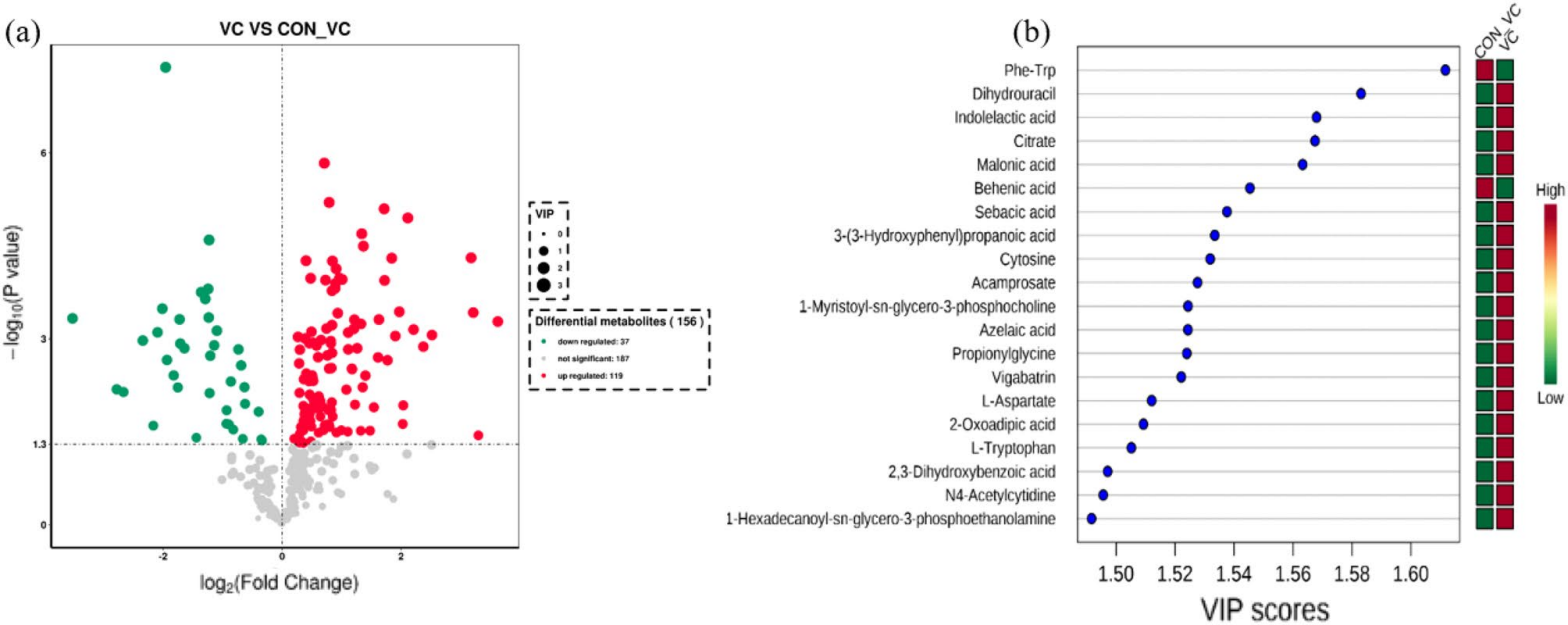
Differentially altered metabolites in blood serum due to VC injection in yak. (**a**) Volcano plots of the difference in metabolite concentrations. Each point in the volcano map represents a metabolite, red and blue dots indicate up-regulated and down-regulated metabolites respectively. Metabolites with no difference between groups are shown in gray. (**b**) Metabolites are ranked by variable importance in projection analysis (VIP) in the respective group. The top 20 important metabolites were arranged from top to bottom according to intracellular concentration. The red box represents a high concentration of the molecule and the green box represents a low concentration.

The effect of VC injection on metabolic profiles in serum are shown in Fig. 5. There were 156 significant differentially altered metabolites at a *P* < 0.05 in response to VC injection (Fig. 5a). VC induced an increase in concentration of 119 metabolites and a decrease in 37 metabolites (Fig. 5a). More details about the differentially altered metabolites are shown in Supplemental Table S2. The top 20 differentially altered metabolites with VIP > 1.5 and *P* < 0.05 are depicted in VIP plots (Fig. 5b). VC injection led to the highest concentrations of Dihydrouracil, Indolelactic acid, Citrate, Malonic acid, Sebacic acid, 3-(3-Hydroxyphenyl) propanoic acid, Cytosine, Acamprosate, 1-Myristoyl-sn-glycero-3-phosphocholine, Azelaic acid, Propionylglycine, Vigabatrin, l-Aspartate, 2-Oxoadipic acid, l-Tryptophan,2,3-Dihydroxybenzoic acid, N4-Acetylcytidine and 1-Hexadecanoyl-sn-glycero-3-phosphoethanolamine. Compared with VC_CON, a decrease in Phe-Trp and Behenic acid were associated with VC injection.

**Figure 6:**
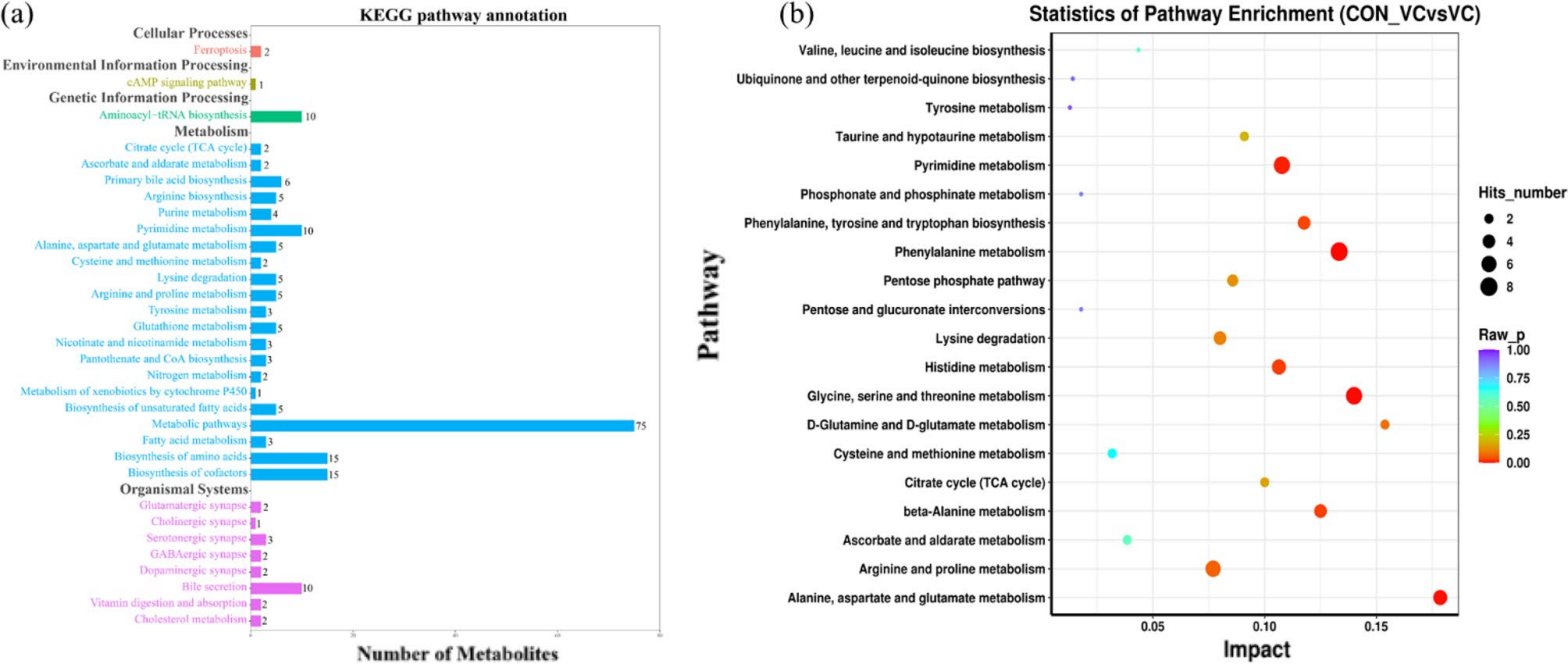
KEGG pathway annotation and enrichment analysis of metabolites in yak serum after VC injection. (**a**) KEGG pathway annotation analysis of metabolic pathways. The horizontal axis is the number of differentially altered metabolites, and the vertical axis is the pathway. Different colors represent different secondary-level of pathway classification in the KEGG system. (**b**) Enrichment analysis of the top 20 metabolic pathways. The color and size of each circle is based on *P*-values and pathway impact values respectively.

**Figure 7:**
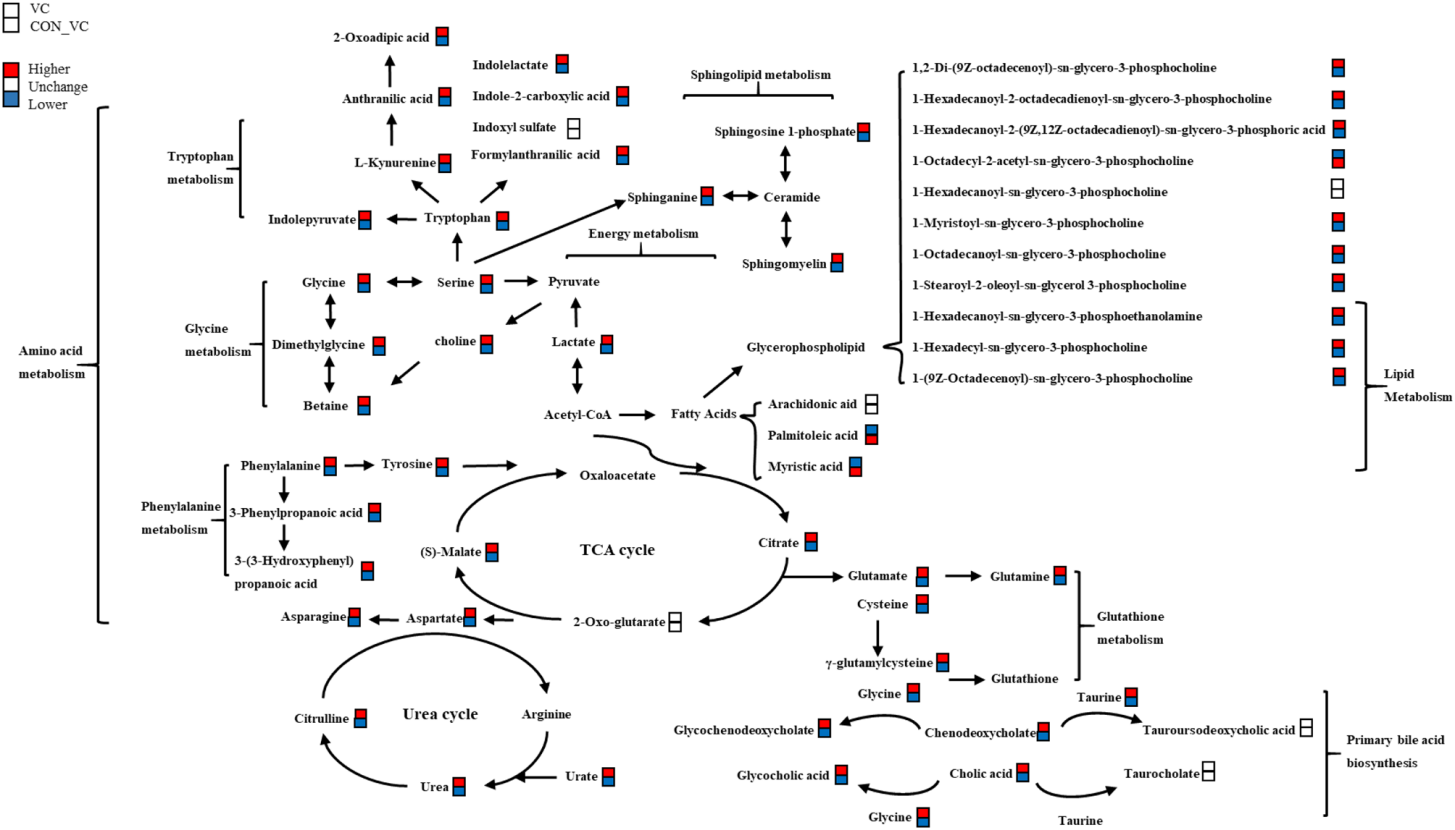
Metabolic network in yak serum induced by VC injection. The red boxes represents the higher concentration of metabolites and the blue boxes represents the lower.

KEGG pathway annotation analysis was performed based on the significant (*P* < 0.05, VIP > 1.0) differentially altered metabolites (Fig. 6a). All of the differentially altered metabolites were classified into cellular processes, environmental information processing, genetic information processing, metabolism and organismal systems (Fig. 6a). VC significantly affected citrate cycle, glutathione, pantothenate and CoA biosynthesis, fatty acid, amino acids biosynthesis and cofactors biosynthesis, all of which are associated with functions that can help reduce oxidative stress. Enrichment pathways with *P* < 0.05 are shown in Fig. 6b. Interestingly, amino acid metabolism had the most stimulation by VC injection. Lastly, a metabolic network (Fig. 7) was created to illustrate the relationships among differentially altered metabolites.

### Correlation analysis between blood metabolites and antioxidants

**Figure 8:**
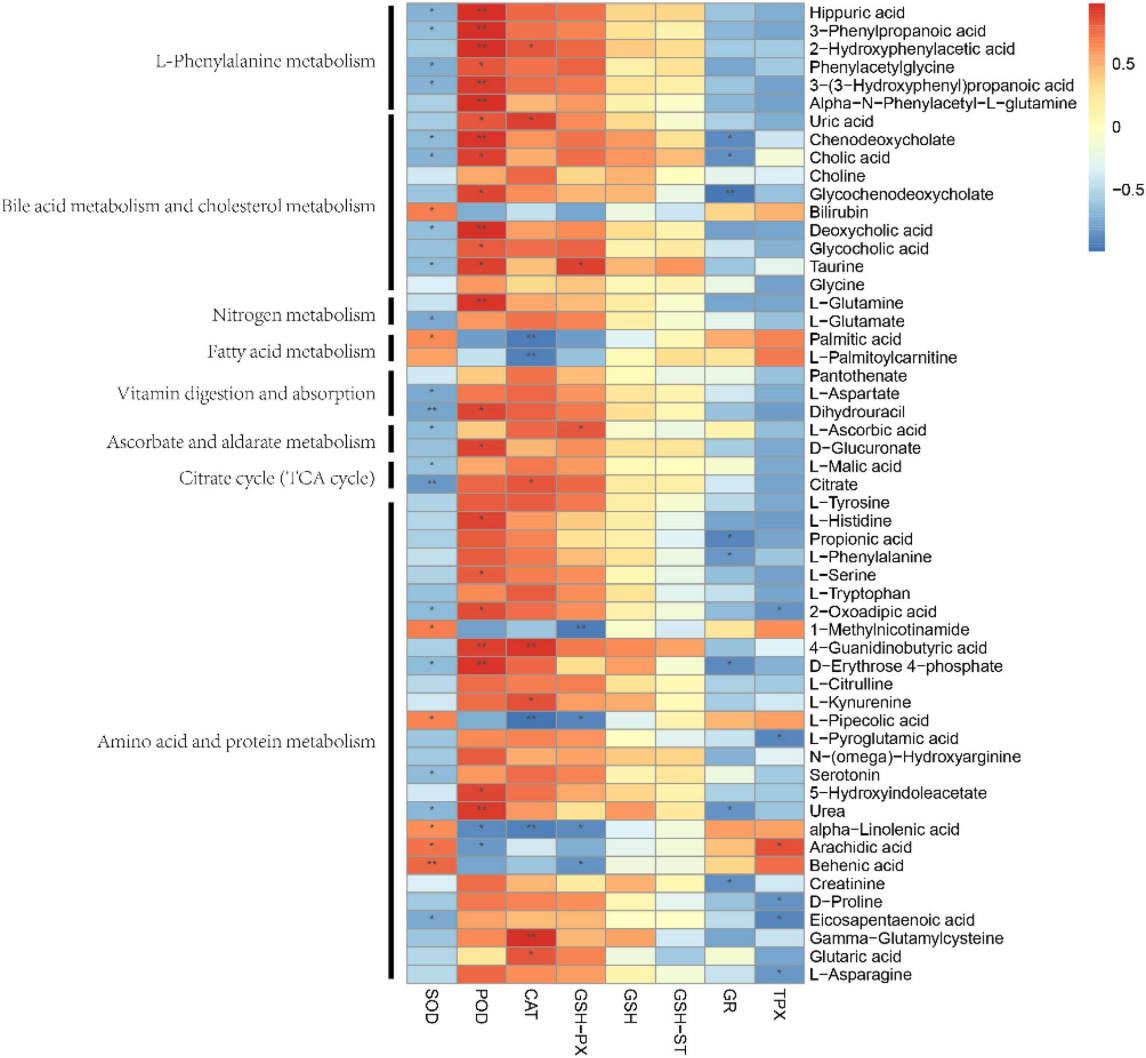
Correlation analysis between blood serum metabolites and antioxidants. The red boxes denote positive correlations and blue boxes negative correlations. *P < 0.05, ** *P* < 0.01.

In order to describe the relationship between blood metabolism and antioxidants, a correlation analysis was performed (Fig. 8). The red color represents a positive correlation, indicating that the metabolite content increased and the antioxidant content increased, while the blue color denotes a negative correlation, indicating that the metabolite content and the antioxidant content were inversely correlated. Amino acid and protein metabolism had the highest number of correlated metabolites, indicating that these pathways had a strong influence on antioxidants and oxidative stress in the yak. SOD had an overall negative correlation, which indicated that the higher the level of metabolites, the lower the activity of this antioxidant enzyme. VC injection caused an increase in the level of some metabolites, which led to a decrease in SOD. In contrast, POD had an overall positive correlation, where an increase in the level of metabolites caused an increase in the level of POD. Similarly, CAT had an overall positive correlation, but the number of related metabolites was not as high as that of POD. Thus, the injection of VC can cause differential changes in metabolism, with the concentration of some molecules potentially affecting the synthesis of antioxidants or activity of antioxidant enzymes in yaks.

## Discussion

### VC serum concentration

VC is one of the most important vitamins with direct functions as anti-oxidant in animals^25^. It regulates oxidative stress through participating in various enzyme reactions associated with the synthesis of amino acids, cholesterol and carnitine^25^. It has been demonstrated that VC injection before transportation increases its concentration in cattle liver and improves the post transit performance^26^. Injections of this vitamin also led to an increase of VC in serum in humans and mice^27,28^. Although ruminants have the capacity to synthesize VC using glucose with the assistance of l-gulonolactone oxidase in liver, the VC level in tissue and blood decreases during stress^29,30^. Thus, it seems plausible that the capacity to synthesize VC in ruminants is diminished during stress conditions. In addition, VC is highly oxidizable and easily dissolve in air. Given the previous experience, VC was immediately injected into yaks within the range of 10–15 g can avoid the dissolution. Although serum glucose content did not differ between VC_CON and VC in this study, the fact that VC concentrations increase due to injection highlighted the usefulness of exogenous VC supplementation in the context of the antioxidant response in yak after transportation.

### Health status of post-transit yak after VC injections

Alanine aminotransferase (ALT), aspartate aminotransferase (AST) and bilirubin (Bil) are important biomarkers to evaluate hepatocyte damage^31^. An increase of blood ALT, AST and T-Bil were observed in dairy cows with postpartal endometritis and mastitis^32^. Oxidative stress is one of the factors that can injure hepatocytes leading to high blood concentrations of ALT and AST^33^, and combined with high T-Bil concentrations, these responses suggest the existence of oxidative stress^34^. Thus, it is plausible that the decrease of ALT, AST and T-Bil concentrations with VC injection reflected an improvement in liver function in post-transit yak.

High concentrations of creatinine (CREA) and UREA are normally used to evaluate kidney injury^35^. Creatinine is used most commonly to assess kidney function, thus the increasing of CREA in blood with VC injection in our study suggested that VC might lead to kidney dysfunction in post-transit yak. But the decreasing of serum UREA level was also observed in our study with VC injection^36^. Moreover, VC supplementation at 100 mg/kg led to lower serum concentrations of CREA and UREA in rats^37^. Given the same supplemented dose of VC with our study, we speculated that VC injection could partly improve renal function, but more work should be performed to elucidate the relationship between VC and the serum level of CREA in yak. C-reactive protein (CRP) is confirmed as a biomarker of inflammation^38^. A positive association between oxidative stress and CRP was reported recently^39^. Thus, it seems plausible that the VC injection reduced inflammation status through the regulation of oxidative stress in yak after transportation.

### Oxidative stress state with VC injection in post-transit yak

LPO, MDA and ROS are normally used to evaluate the state of oxidative stress in animals and humans. Both LPO and MDA are biomarkers of lipid peroxidation^40^. MDA is the final product of lipid peroxidation and can cause cell damage^41^. There is a positive relationship between serum level of MDA and intensity of oxidative stress^40^. Thus, our data of higher serum level of MDA after VC injection suggested that it may have promoted oxidative stress in post-transit yak through stimulating lipid peroxidation. The reason for promoting lipid peroxidation may be semi-dehydroascorbic (A^-^), a product of VC promoting oxidation. When A^-^ cannot be cleared in a timely manner, lipid peroxidation occurs, causing an increase in MDA. However, no significant difference in LPO was observed in the present study, which is the primary oxidation production once the peroxidation reaction begins^42^. Furthermore, it is noteworthy that MDA is the product of reducing hydrogen peroxide (H_2_O_2_) to water using VC with the assistance of ascorbate peroxidase^43^. It has been demonstrated that MDA not only causes cell damage during oxidative stress, but also functions as the biologically active compound^44^. Furthermore, no difference in ROS was observed in our study. Thus, combined with the higher serum level of VC, we speculated that VC injection had the ability to potentially alleviated oxidative stress state through regulating MDA synthesis in post-transit yaks.

### VC improves the anti-oxidant capacity in post-transit yaks

Oxidative stress is induced because of the imbalance between ROS accumulation and antioxidant defenses. To further evaluate the influence of VC injection on anti-oxidant capacity in post-transit yak, the determination of serum levels of antioxidants was performed. H_2_O_2_ is a central redox molecule for the reduction to produce H_2_O and O_2_ with the assistance of GSH-PX and CAT^45^. Thus, our data of greater serum level of GSH-PX and CAT suggested that VC injection could promote anti-oxidant capacity in post-transit yaks.

Activity of SOD is the first line of defense against oxidative stress through catalyzing superoxide anion radical (O_2_^**·**−^) to H_2_O_2_^46^. Oxidative stress in the body may be related to superoxide anion, hydroxyl free radical, and hydrogen peroxide. Individual and environmental differences may lead to different levels of oxidative stress markers, which may lead to differences in the content of corresponding antioxidant enzymes. Therefore, the reason for the decrease in SOD level may be the decrease of superoxide anion content in yaks after VC injection. Interestingly, VC injection led to greater serum level of POD, which could directly promote the generation of H_2_O_2_ without the oxidative reaction of O_2_ to superoxide anion radical (O_2_^**·**−^)^47^. Given that the same product (H_2_O_2_) of the catalyzing reaction of SOD and POD, it seems plausible that competition between the SOD and POD systems occurred relative to the generation of H_2_O_2_. Similar results were also observed in previous studies^48,49^. Thus, we speculated that VC injection could improve the anti-oxidant capability of post-transit yak through increasing the serum level of POD to regulate H_2_O_2_ metabolism.

### Metabolomics

This study has demonstrated that VC injections can cause significant changes in metabolic pathways, with amino acid metabolism being the most stimulated by the VC injection. The present study has also demonstrated that VC injections can alter the synthesis of GSH through regulation of glucose metabolism.

#### Glutathione metabolism

Glutathione plays an important role in the protection of cells from oxidative damage, a process which can cause cell death^50^. Glutathione is a non-protein sulfhydryl tripeptide compound formed by the condensation of glutamic acid, cysteine, and glycine through peptide bonds^51^. Thus, changes in the blood concentrations of these three amino acids can affect the concentration of glutathione. Glutamate is located at the center of ammonia–nitrogen exchange and is a major carrier of most non-essential amino acids such as ornithine and citrulline. It is an important precursor for the synthesis of nucleotides, amino sugars, and nicotinamide adenosine dinucleotides. Increased levels of glutamine can be catabolized to compensate for the loss of alpha-ketoglutarate and replenished into the TCA cycle, maintaining adenosine triphosphate and glutathione levels in response to oxidative stress^52,53^. The injection of VC increased glutamate concentrations, and there was an association between glutamate concentrations and antioxidants. In addition, the concentration of glutamate was negatively correlated with SOD, GSH-ST, and TPX, and positively correlated with CAT, POD, and GSH-PX, with the greatest correlation with CAT. Thus, together, these data indicated that an increase in glutamate concentrations could have been responsible for the increase in CAT. The results of the analysis are consistent with the changes in antioxidant concentrations we tested, thus, we speculated that the injection of VC enhanced antioxidant capacity through glutamate.

Cysteine is an amino acid that is commonly found in living organisms. Methionine and cysteine are used as precursors for S-adenosylmethionine, taurine, sulfuric acid, and glutathione^54^. In the present study, VC injection caused an increase in cysteine levels, suggesting that VC promoted activity of the one-carbon metabolism pathway to enhance the metabolism of methionine to homocysteine, which was then rapidly converted to cystathionine. Subsequently, this molecule was converted to taurine and glutathione via the transculturation pathway to mitigate oxidative stress due to transportation. Glycine is a non-essential amino acid that is used in the endogenous production of glutathione, and is often supplemented exogenously when stress occurs in the organism^55^. In this study, VC injection caused an increase of glycine levels suggesting that glycine levels play an important role in the alleviation of oxidative stress.

Besides the increase in the concentrations of glutamate, cysteine, and glycine, and the results of KEGG pathway annotation, VC injections increased glutathione concentrations. Thus, we hypothesized that a key effect of VC injection is to trigger various mechanisms to enhance glutathione synthesis to restore antioxidant capacity during transportation stress.

#### Phenylalanine, tyrosine and tryptophan metabolism

Phenylalanine can be metabolized to tyrosine via the enzyme phenylalanine hydroxylase. This increase in essential amino acid content induces oxidative stress^56^. The reported effect of phenylalanine on the expression or activity of enzymes participating in metabolic pathways known to be responsive to redox signaling might be mediated through oxidative stress^57^. Therefore, we hypothesize that the increase in phenylalanine levels is related to the transport stress status of yaks. At the same time, the increase in phenylalanine levels also caused an increase in tyrosine levels. Despite the overwhelming evidence for an association between phenylalanine and oxidative stress, it remains unclear whether VC injection is a determinant of the rise in phenylalanine levels and further development of research in this area is needed.

Tryptophan plays (an essential amino acid) an important role in the metabolism, development and growth of animals^58^, and it also participates in regulating immunity of livestock^59^. Tryptophan is a precursor of active molecules such as melatonin, a metabolite with antioxidant effects. In addition, 2-Oxoadipic acid, a key metabolite of tryptophan, during the metabolism of tryptophan, the content of 2-Oxoadipic acid was significantly increased in the VC injection group. This is consistent with the higher serum concentration of 2-Oxoadipic acid determined based on VIP analysis. In the correlation analysis, 2-Oxoadipic acid, which is involved in tryptophan metabolism as a member of the amino acid metabolic process, showed positive correlation with SOD and POD, indicating a close relationship between tryptophan metabolism and antioxidants. This conclusion is consistent with the study of Yao et al. who showed that tryptophan and its metabolites (e.g., serotonin (5-hydroxytryptamine, 5-HT) and melatonin)) can regulate feed intake, reproduction, immunity, neurological function, and anti-stress responses^60^. Therefore, it is reasonable to speculate that the increased tryptophan metabolism induced by VC injection is effective against oxidative stress.

#### Alanine and aspartate metabolism

Transamination plays an important role in the degradation of alanine, aspartate and glutamate. This process results in the production of pyruvate, oxaloacetate, and alpha ketoglutarate, which can serve as carbon sources for the tricarboxylic acid cycle and a source of adenosine triphosphate for the synthesis of purine and pyrimidine nucleotides^61^. Thus, the increased concentrations of glutamine, citrate, and aspartate after VC injection indicated that transamination of alanine, aspartate, and glutamate was increased to provide a carbon source for the TCA cycle.

The correlation analysis revealed a relationship between choline and various antioxidants. It shows a negative correlation with SOD, GR, and TPX, and a positive correlation with POD, CAT, GSH-PX, GSH, and GSH-ST, suggesting that an increase in choline concentration caused a decrease in SOD, GR, and TPX concentrations and an increase in the concentrations of other antioxidants that were positively correlated with choline. The changes caused by choline were less pronounced because the correlation was not very strong, which is consistent with the results of our tests on the changes in antioxidant content. Thus, it can be hypothesized that VC injection caused an upregulation of alanine concentration, which in turn led to an increase in pyruvate and choline concentrations to regulate antioxidant levels.

The concentration of asparagine was increased after VC injection. The results of the correlation analysis showed that asparagine also had relationships with various types of antioxidants; it had a significant negative correlation with SOD, GR, and TPX, and a significant positive correlation with POD, CAT, and GSH-PX, and when the concentration of asparagine increased, the concentration of POD, CAT, and GSH-PX also increased, and conversely, the concentration of SOD, GR, and TPX decreased. This is consistent with the results of the tests we did on the variation of antioxidant concentration. Thus, it can be inferred that the injection of VC caused the change of aspartate metabolism, which led to the upregulation of asparagine concentration and finally caused the change of antioxidant content. In our study, changes in aspartate content could modulate the antioxidant content, a finding consistent with that of Sivaperumal et al.^62^. In their study, aspartic acid could regulate lipid peroxidation as well as antioxidants. This suggests that the injection of VC can protect against oxidative stress by affecting the metabolism and thus the oxidative stress.

## Conclusion

In the present study, our data provided more systematic evidence based on metabolomics and antioxidative expression that VC injections are able to modulate antioxidant levels by affecting differential changes in metabolism to resist oxidative stress generated by yaks during transport. Considering the increased concentrations of most essential amino acids within the serum, future studies may focus on the requirement of vitamin C for essential amino acids regulation. Also, future studies may focus on the appropriate dose of vitamins C to anti-oxidative stress regulation.

## Supplementary Information

Supplementary Tables.

## Data Availability

The datasets generated during and/or analysed during the current study are available from the corresponding author on reasonable request.
