# Supplementary material for: Vitamin C injection improves antioxidant stress capacity through regulating blood metabolism in post-transit yak
Source: Sci Rep. 2023 Jun 23;13:10233. doi: 10.1038/s41598-023-36779-w (PMC10290073; doi:10.1038/s41598-023-36779-w)
Supplement: Supplementary file 1 — Supplementary Tables. [file 41598_2023_36779_MOESM1_ESM.docx]

**Supplementary Information**

**Supplemental Table 1 The results of identifiable** **metabolites**

| Items | Total metabolites | Differential metabolites | MS2 score |
| --- | --- | --- | --- |
| amount | 343 | 156 | 0.7944 |

**Supplemental Table 2 The concentration of metabolites in the CON_VC&VC groups**

| Metabolites | VIP | *P*-Value | UP/DOWN |
| --- | --- | --- | --- |
| Phe-Trp | 1.6117 | <0.0001 | DOWN |
| Dihydrouracil | 1.5831 | <0.0001 | UP |
| Indolelactic acid | 1.5680 | <0.0001 | UP |
| Citrate | 1.5675 | <0.0001 | UP |
| Malonic acid | 1.5633 | <0.0001 | UP |
| Behenic acid | 1.5454 | <0.0001 | DOWN |
| Sebacic acid | 1.5376 | <0.0001 | UP |
| 3-(3-Hydroxyphenyl)propanoic acid | 1.5335 | <0.0001 | UP |
| Cytosine | 1.5319 | 0.0001 | UP |
| Acamprosate | 1.5276 | <0.0001 | UP |
| Azelaic acid | 1.5244 | <0.0001 | UP |
| 1-Myristoyl-sn-glycero-3-phosphocholine | 1.5244 | 0.0001 | UP |
| Propionylglycine | 1.5240 | 0.0001 | UP |
| Vigabatrin | 1.5221 | 0.0001 | UP |
| L-Aspartate | 1.5121 | 0.0001 | UP |
| 2-Oxoadipic acid | 1.5093 | 0.0001 | UP |
| Oleic acid / trans-Vaccenic acid | 1.5084 | 0.0002 | DOWN |
| L-Tryptophan | 1.5052 | 0.0002 | UP |
| cis-9-Palmitoleic acid | 1.5025 | 0.0002 | DOWN |
| "2,3-Dihydroxybenzoic acid" | 1.4972 | 0.0001 | UP |
| N4-Acetylcytidine | 1.4957 | 0.0001 | UP |
| 1-Hexadecanoyl-sn-glycero-3-phosphoethanolamine | 1.4917 | 0.0001 | UP |
| Allantoin | 1.4907 | 0.0002 | UP |
| alpha-Linolenic acid | 1.4772 | 0.0002 | DOWN |
| Palmitic acid | 1.4667 | 0.0003 | DOWN |
| Arachidic acid | 1.4638 | 0.0005 | DOWN |
| L-Glutamate | 1.4610 | 0.0004 | UP |
| "3,4-Dihydroxybenzoate (Protocatechuic acid)" | 1.4606 | 0.0005 | UP |
| 1-Methylnicotinamide | 1.4557 | 0.0005 | DOWN |
| 4-Hydroxy-3-methylbenzoic acid | 1.4550 | 0.0005 | UP |
| 3-Phenylpropanoic acid | 1.4534 | 0.0005 | UP |
| L-homocysteic acid | 1.4527 | 0.0004 | UP |
| 1-(9Z-Octadecenoyl)-sn-glycero-3-phosphocholine | 1.4509 | 0.0006 | UP |
| Tridecanoic acid (Tridecylic acid) | 1.4491 | 0.0007 | DOWN |
| L-Pyroglutamic acid | 1.4480 | 0.0007 | UP |
| DL-Norvaline | 1.4436 | 0.0005 | DOWN |
| Hippuric acid | 1.4381 | 0.0007 | UP |
| Trimethylamine N-oxide | 1.4379 | 0.0004 | UP |
| D-Glucuronate | 1.4364 | 0.0008 | UP |
| "1,2-Di-(9Z-octadecenoyl)-sn-glycero-3-phosphocholine" | 1.4360 | 0.0008 | UP |
| Arg-Tyr-Arg | 1.4326 | 0.0006 | UP |
| "3,4-Dihydroxyphenylacetic acid" | 1.4268 | 0.0009 | UP |
| Estrone-3-glucuronide | 1.4268 | 0.0011 | DOWN |
| Cytidine | 1.4254 | 0.0007 | UP |
| D-Proline | 1.4240 | 0.0011 | UP |
| 1-Hexadecyl-sn-glycero-3-phosphocholine | 1.4232 | 0.0012 | UP |
| Glycocholic acid | 1.4197 | 0.0010 | UP |
| L-Tyrosine | 1.4187 | 0.0013 | UP |
| 1-Octadecyl-2-acetyl-sn-glycero-3-phosphocholine | 1.4187 | 0.0012 | DOWN |
| L-Malic acid | 1.4179 | 0.0014 | UP |
| Betaine | 1.4177 | 0.0009 | UP |
| L-Pipecolic acid | 1.4174 | 0.0008 | DOWN |
| Barbituric acid | 1.4145 | 0.0010 | UP |
| Deoxycholic acid | 1.4139 | 0.0012 | UP |
| Leu-Lys-Arg | 1.4027 | 0.0015 | DOWN |
| Serotonin | 1.4017 | 0.0015 | UP |
| Leu-Gln-Arg | 1.4010 | 0.0009 | UP |
| "1-Hexadecanoyl-2-(9Z,12Z-octadecadienoyl)-sn-glycero-3-phosphoric acid" | 1.4008 | 0.0012 | UP |
| Biliverdin | 1.3986 | 0.0014 | DOWN |
| Hydroxyphenyllactic acid | 1.3963 | 0.0012 | UP |
| L-Serine | 1.3947 | 0.0017 | UP |
| L-Norleucine | 1.3895 | 0.0013 | DOWN |
| Sphinganine | 1.3892 | 0.0022 | UP |
| Uric acid | 1.3835 | 0.0020 | UP |
| Dimethyl sulfone | 1.3816 | 0.0015 | UP |
| Indole-2-carboxylic acid | 1.3768 | 0.0013 | UP |
| "1,4-Dihydroxybenzene" | 1.3746 | 0.0020 | UP |
| Myristic acid | 1.3744 | 0.0022 | DOWN |
| Suberylglycine | 1.3742 | 0.0019 | DOWN |
| L-Asparagine | 1.3735 | 0.0030 | UP |
| Edaravone | 1.3649 | 0.0018 | UP |
| Gly-Glu | 1.3591 | 0.0027 | DOWN |
| 1-Methylhistamine | 1.3579 | 0.0025 | UP |
| 2-Hydroxyphenylacetic acid | 1.3505 | 0.0030 | UP |
| Alpha-N-Phenylacetyl-L-glutamine | 1.3427 | 0.0031 | UP |
| 6-Phospho-D-gluconate | 1.3427 | 0.0030 | UP |
| (S)-2-Hydroxyglutarate | 1.3326 | 0.0039 | UP |
| Glutaric acid | 1.3276 | 0.0045 | UP |
| Benzoic acid | 1.3249 | 0.0039 | UP |
| L-Citrulline | 1.3235 | 0.0047 | UP |
| L-Histidine | 1.3207 | 0.0042 | UP |
| Coniferyl aldehyde | 1.3204 | 0.0039 | UP |
| N-.alpha.-Acetyl-L-ornithine | 1.3196 | 0.0039 | DOWN |
| D-Erythrose 4-phosphate | 1.3151 | 0.0036 | UP |
| L-Palmitoylcarnitine | 1.3129 | 0.0049 | DOWN |
| "15-Deoxy-delta-12,14-PGJ2" | 1.3040 | 0.0065 | DOWN |
| sn-Glycerol 3-phosphoethanolamine | 1.2950 | 0.0085 | UP |
| N-Acetyl-L-tyrosine | 1.2907 | 0.0048 | UP |
| Hexacosanoic acid | 1.2800 | 0.0075 | DOWN |
| Citraconic acid | 1.2799 | 0.0066 | UP |
| Bilirubin | 1.2734 | 0.0061 | DOWN |
| Glycochenodeoxycholate | 1.2697 | 0.0060 | UP |
| DL-Indole-3-lactic acid | 1.2681 | 0.0060 | DOWN |
| L-Glutamine | 1.2663 | 0.0093 | UP |
| D-Fructose | 1.2594 | 0.0102 | UP |
| Curcumin | 1.2552 | 0.0092 | UP |
| Trp-Gly-Lys | 1.2486 | 0.0072 | DOWN |
| Pantothenate | 1.2469 | 0.0106 | UP |
| 1-Octadecanoyl-sn-glycero-3-phosphocholine | 1.2464 | 0.0124 | UP |
| Eicosapentaenoic acid | 1.2392 | 0.0121 | UP |
| L-Phenylalanine | 1.2381 | 0.0101 | UP |
| Pseudouridine | 1.2375 | 0.0079 | UP |
| Gamma-Glutamylcysteine | 1.2327 | 0.0074 | UP |
| Adipic acid | 1.2314 | 0.0124 | UP |
| 3-Hydorxy-3-methylglutaric acid | 1.2274 | 0.0130 | UP |
| L-Ascorbic acid | 1.2266 | 0.0127 | UP |
| Formylanthranilic acid | 1.2187 | 0.0141 | UP |
| L-Kynurenine | 1.2178 | 0.0133 | UP |
| Acetylcarnitine | 1.2070 | 0.0111 | DOWN |
| Glycodeoxycholic acid | 1.2015 | 0.0115 | UP |
| Chenodeoxycholate | 1.1966 | 0.0118 | UP |
| 1-Phenoxy-2-propanol | 1.1966 | 0.0142 | DOWN |
| Capric acid | 1.1945 | 0.0149 | DOWN |
| Sphingomyelin (d18:1/18:0) | 1.1902 | 0.0163 | UP |
| Taurine | 1.1822 | 0.0141 | UP |
| Deoxycytidine | 1.1788 | 0.0161 | UP |
| Phenylacetylglycine | 1.1740 | 0.0156 | UP |
| Urea | 1.1719 | 0.0179 | UP |
| Choline | 1.1714 | 0.0200 | UP |
| Indole-3-pyruvic acid | 1.1571 | 0.0233 | UP |
| DL-Arginine | 1.1493 | 0.0212 | UP |
| Dimethylglycine | 1.1455 | 0.0204 | UP |
| 16-Hydroxypalmitic acid | 1.1410 | 0.0234 | DOWN |
| Lys-Gln-Arg | 1.1398 | 0.0179 | UP |
| 3-Hydroxydodecanoic acid | 1.1385 | 0.0251 | DOWN |
| Dimetridazole | 1.1375 | 0.0248 | UP |
| Anthranilic acid (Vitamin L1) | 1.1355 | 0.0263 | UP |
| 4-Imidazoleacetic acid | 1.1353 | 0.0236 | UP |
| Glycolithocholic acid | 1.1349 | 0.0178 | UP |
| N-Palmitoyltaurine | 1.1335 | 0.0239 | DOWN |
| DL-2-Phosphoglycerate | 1.1269 | 0.0242 | UP |
| 5-Hydroxyindoleacetate | 1.1263 | 0.0296 | UP |
| N-(omega)-Hydroxyarginine | 1.1237 | 0.0212 | UP |
| DL-lactate | 1.1236 | 0.0302 | UP |
| 1-Stearoyl-2-oleoyl-sn-glycerol 3-phosphocholine (SOPC) | 1.1187 | 0.0274 | UP |
| Enterostatin human | 1.1152 | 0.0297 | UP |
| Propionic acid | 1.1095 | 0.0264 | UP |
| Thymidine | 1.1074 | 0.0291 | DOWN |
| Asn-Val-Arg | 1.1060 | 0.0239 | UP |
| D-erythro-Sphingosine-1-phosphate | 1.1043 | 0.0298 | UP |
| 2'-O-methylcytidine | 1.1015 | 0.0260 | UP |
| Phe-Thr | 1.0961 | 0.0409 | DOWN |
| 4-Guanidinobutyric acid | 1.0923 | 0.0325 | UP |
| 1-Hexadecanoyl-2-octadecadienoyl-sn-glycero-3-phosphocholine | 1.0889 | 0.0326 | UP |
| Glycine | 1.0747 | 0.0476 | UP |
| Ribothymidine | 1.0715 | 0.0360 | UP |
| Lys-Ile-Arg | 1.0692 | 0.0303 | UP |
| 1-Naphthol | 1.0686 | 0.0452 | UP |
| Triethylene glycol | 1.0650 | 0.0343 | UP |
| Cholic acid | 1.0610 | 0.0314 | UP |
| Dodecanoic acid | 1.0547 | 0.0436 | DOWN |
| "1,2-Benzenedicarboxylic acid" | 1.0529 | 0.0408 | UP |
| Stearoylcarnitine | 1.0472 | 0.0392 | DOWN |
| gamma-L-Glutamyl-L-valine | 1.0383 | 0.0472 | UP |
| Creatinine | 1.0290 | 0.0448 | UP |
| D-Glucosaminic acid | 1.0288 | 0.0416 | DOWN |
